# 1-(4-Methoxy­phen­yl)-3-methyl-1*H*-1,2,4-triazol-5(4*H*)-one

**DOI:** 10.1107/S1600536808031784

**Published:** 2008-10-11

**Authors:** Bing Liu, Weiren Xu, Guilong Zhao, Runling Wang, Lida Tang

**Affiliations:** a School of Pharmacy, Tianjin Medical University, Tianjin 300070, People’s Republic of China; b Tianjin Key Laboratory of Molecular Design and Drug Discovery, Tijin Institute of Pharmaceutical Research, Tianjin 300193, People’s Republic of China; c Tianjin Key Laboratory of Pharmacokinetics and Pharmacodynamics, Tijin Institute of Pharmaceutical Research, Tianjin 300193, People’s Republic of China

## Abstract

In the title compound, C_10_H_11_N_3_O_2_, the triazole ring has a dihedral angle of 11.55 (2)° relative to the benzene ring. The crystal packing is stabilized by inter­molecular N—H⋯O and C—H⋯O hydrogen bonds, and by weak π–π stacking inter­actions [centroid-to-centroid distance = 3.545 (3) Å].

## Related literature

For related literature on the biological activity of the title compound, see: Kitazaki *et al.* (1996 ▶); John (1996 ▶). For reference structural data, see: Allen *et al.* (1987 ▶).
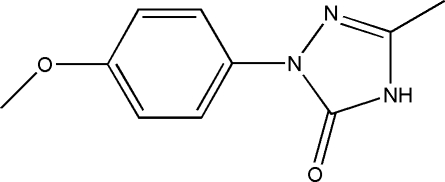


## Experimental

### 

#### Crystal data

C_10_H_11_N_3_O_2_
*M* *_r_* = 205.22
Orthorhombic, 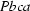

*a* = 13.244 (3) Å
*b* = 8.4865 (17) Å
*c* = 17.518 (4) Å
*V* = 1968.9 (7) Å^3^
*Z* = 8
Mo *K*α radiation
μ = 0.10 mm^−1^
*T* = 113 (2) K
0.16 × 0.14 × 0.12 mm

#### Data collection

Rigaku Saturn diffractometer
Absorption correction: multi-scan (*CrystalClear*; Rigaku, 2005 ▶) *T*_min_ = 0.973, *T*_max_ = 0.988
12523 measured reflections
2163 independent reflections
1923 reflections with *I* > 2σ(*I*)
*R*_int_ = 0.046

#### Refinement

*R*[*F* ^2^ > 2σ(*F* ^2^)] = 0.045
*wR*(*F* ^2^) = 0.115
*S* = 1.09
2163 reflections
143 parameters
H atoms treated by a mixture of independent and constrained refinement
Δρ_max_ = 0.19 e Å^−3^
Δρ_min_ = −0.21 e Å^−3^

### 

Data collection: *CrystalClear* (Rigaku, 2005 ▶); cell refinement: *CrystalClear*; data reduction: *CrystalClear*; program(s) used to solve structure: *SHELXTL* (Sheldrick, 2008 ▶); program(s) used to refine structure: *SHELXTL*; molecular graphics: *SHELXTL*; software used to prepare material for publication: *SHELXTL*.

## Supplementary Material

Crystal structure: contains datablocks I, global. DOI: 10.1107/S1600536808031784/sg2268sup1.cif

Structure factors: contains datablocks I. DOI: 10.1107/S1600536808031784/sg2268Isup2.hkl

Additional supplementary materials:  crystallographic information; 3D view; checkCIF report

## Figures and Tables

**Table 1:** Hydrogen-bond geometry (Å, °)

*D*—H⋯*A*	*D*—H	H⋯*A*	*D*⋯*A*	*D*—H⋯*A*
C10—H10*C*⋯O1^i^	0.96	2.57	3.4918 (18)	160
N1—H1⋯O1^ii^	0.938 (18)	1.825 (19)	2.7561 (16)	171.9 (16)

Symmetry codes: (i) 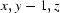; (ii) 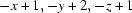.

**Fig. 1.**
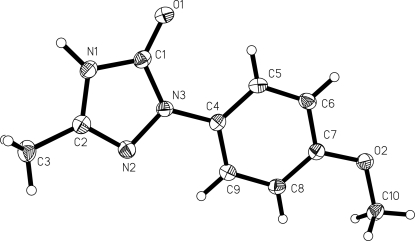
View of the title compound (I), with displacement ellipsoids drawn at the 40% probability level.

**Fig. 2.**
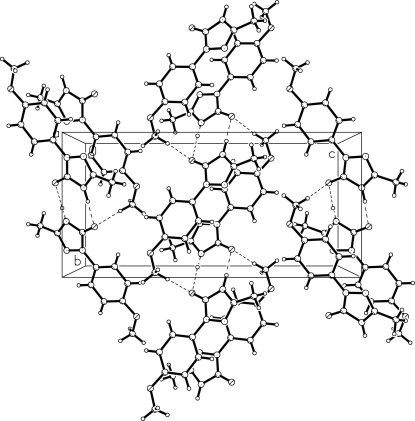
A packing diagram of the molecule of the title compound, viewed down a axis. Hydrogen bonds are shown as dashed lines.

## supplementary crystallographic information

### Comment

1-Aryl-1,5-dihydro-1,2,4-triazol-5-ones are a class of important intermediates in the synthesis of some biologically active compounds (Kitazaki *et al.*, 1996; John, 1996). In our effort to study this class of compounds as anticancer agents, the title compound (I) was prepared as an important intermediate.

In (I) (Fig. 1), all bond lengths are normal (Allen *et al.*, 1987).The triazole ring (C1/C2/N1/N2/N3) make a dihedral angles of 11.55 (2)° with the phenyl ring (C4—C9). The crystal packing is stabilized by intermolecular N—H···O and C—H···O hydrogen bonds. The relatively short distance of 3.545 (3) between the centroids of benzene ring C4—C9 and triazole ring C1/C2/N1/N2/N3 [at -*x*,1 - *y*,-*z*] indicates the presence of weak π-π interactions, which contribute to the stability of the crystal packing.

### Experimental

Pyruvic acid (2.21 g, 25 mmol) was added to a 20 ml of aqueous solution of (4-Methoxyphenyl)hydrazine hydrochloride (4.0 g, 23 mmol). The  solution was stirred for 1 h. The precipitate was collected by filtration, washed with water and dried over P_2_O_5_ to give 2-[(4-Methoxyphenyl) hydrazono]propionic acid (3.45 g, yield 72.4%) as a yellow powder. 2-[(4-Methoxyphenyl)-hydrazono]propionic acid (3.45 g, 16.5 mmol) was suspended in toluene, and triethylamine (1.67 g, 16.5 mmol) and diphenylphosphoryl azide [(PHO)_2_PON_3_, 4.5 g, 16.5 mmol] were added to the suspension. The resulting mixture was heated at 120 ° C for 3 h. It was then cooled and extracted with 10% aqueous NaOH (30 ml). The aqueous extract was acidified (to pH = 1) with concentrated HCl. The crystals were collected by filtration and recrystallized from CH_3_CN to give the title compound (2.8 g, 82%) as a colorless power. The single-crystal suitable for X-ray diffraction was obtained by slow evaporation of a solution of the title compound in CH_2_Cl_2_ and cyclohexane (V:V 1:1).

### Refinement

All H atoms were found in difference maps. The N—H atoms were refined freely, giving an N—H bond distance of 0.94 Å. The remaining H atoms were placed in calculated positions, with C—H = 0.93 or 0.96 Å, and included in the final cycles of refinement using a riding model, with *U*_iso_(H) = 1.2 (1.5 for methyl) times *U*_eq_(C).

### Crystal data

**Table:**

C_10_H_11_N_3_O_2_	*F*(000) = 864
*M**_r_* = 205.22	*D*_x_ = 1.385 Mg m^−^^3^
Orthorhombic, *P**b**c**a*	Mo *K*α radiation, λ = 0.71073 Å
Hall symbol: -P 2ac 2ab	Cell parameters from 4443 reflections
*a* = 13.244 (3) Å	θ = 1.5–27.1°
*b* = 8.4865 (17) Å	µ = 0.10 mm^−^^1^
*c* = 17.518 (4) Å	*T* = 113 K
*V* = 1968.9 (7) Å^3^	Block, colorless
*Z* = 8	0.16 × 0.14 × 0.12 mm

### Data collection

**Table:**

Rigaku Saturn diffractometer	2163 independent reflections
Radiation source: rotating anode	1923 reflections with *I* > 2σ(*I*)
confocal	*R*_int_ = 0.046
Detector resolution: 7.31 pixels mm^-1^	θ_max_ = 27.1°, θ_min_ = 2.8°
ω scans	*h* = −16→16
Absorption correction: multi-scan (Crystalclear; Rigaku, 2005)	*k* = −10→10
*T*_min_ = 0.973, *T*_max_ = 0.988	*l* = −19→22
12523 measured reflections	

### Refinement

**Table:**

Refinement on *F*^2^	Secondary atom site location: difference Fourier map
Least-squares matrix: full	Hydrogen site location: inferred from neighbouring sites
*R*[*F*^2^ > 2σ(*F*^2^)] = 0.045	H atoms treated by a mixture of independent and constrained refinement
*wR*(*F*^2^) = 0.115	*w* = 1/[σ^2^(*F*_o_^2^) + (0.0626*P*)^2^ + 0.2559*P*] where *P* = (*F*_o_^2^ + 2*F*_c_^2^)/3
*S* = 1.09	(Δ/σ)_max_ < 0.001
2163 reflections	Δρ_max_ = 0.19 e Å^−^^3^
143 parameters	Δρ_min_ = −0.21 e Å^−^^3^
0 restraints	Extinction correction: SHELXTL (Sheldrick, 2008), Fc^*^=kFc[1+0.001xFc^2^λ^3^/sin(2θ)]^-1/4^
Primary atom site location: structure-invariant direct methods	Extinction coefficient: 0.010 (2)

### Special details

**Table:**

Geometry. All e.s.d.'s (except the e.s.d. in the dihedral angle between two l.s. planes) are estimated using the full covariance matrix. The cell e.s.d.'s are taken into account individually in the estimation of e.s.d.'s in distances, angles and torsion angles; correlations between e.s.d.'s in cell parameters are only used when they are defined by crystal symmetry. An approximate (isotropic) treatment of cell e.s.d.'s is used for estimating e.s.d.'s involving l.s. planes.
Refinement. Refinement of *F*^2^ against ALL reflections. The weighted *R*-factor *wR* and goodness of fit *S* are based on *F*^2^, conventional *R*-factors *R* are based on *F*, with *F* set to zero for negative *F*^2^. The threshold expression of *F*^2^ > σ(*F*^2^) is used only for calculating *R*-factors(gt) *etc*. and is not relevant to the choice of reflections for refinement. *R*-factors based on *F*^2^ are statistically about twice as large as those based on *F*, and *R*- factors based on ALL data will be even larger.

### Fractional atomic coordinates and isotropic or equivalent isotropic displacement parameters (Å^2^)

**Table:**

	*x*	*y*	*z*	*U*_iso_*/*U*_eq_	
O1	0.50756 (8)	0.83446 (11)	0.57225 (5)	0.0310 (3)	
O2	0.39300 (8)	0.14256 (11)	0.72040 (6)	0.0292 (3)	
N1	0.41360 (9)	0.87270 (14)	0.46051 (6)	0.0263 (3)	
N2	0.33167 (9)	0.64616 (13)	0.45502 (6)	0.0252 (3)	
N3	0.39458 (9)	0.65123 (13)	0.51918 (6)	0.0229 (3)	
C1	0.44607 (11)	0.79085 (16)	0.52321 (7)	0.0249 (3)	
C2	0.34610 (11)	0.78137 (16)	0.42171 (7)	0.0249 (3)	
C3	0.29407 (12)	0.83520 (17)	0.35153 (8)	0.0328 (4)	
H3A	0.2456	0.7574	0.3361	0.049*	
H3B	0.3427	0.8500	0.3115	0.049*	
H3C	0.2602	0.9331	0.3615	0.049*	
C4	0.39649 (10)	0.52033 (15)	0.56989 (7)	0.0219 (3)	
C5	0.44275 (11)	0.53031 (16)	0.64158 (7)	0.0253 (3)	
H5	0.4753	0.6224	0.6566	0.030*	
C6	0.43944 (11)	0.40130 (17)	0.68989 (7)	0.0264 (3)	
H6	0.4695	0.4077	0.7378	0.032*	
C7	0.39175 (10)	0.26195 (15)	0.66796 (7)	0.0234 (3)	
C8	0.34760 (10)	0.25219 (16)	0.59617 (7)	0.0248 (3)	
H8	0.3163	0.1594	0.5807	0.030*	
C9	0.35032 (10)	0.38157 (16)	0.54756 (7)	0.0242 (3)	
H9	0.3208	0.3748	0.4995	0.029*	
C10	0.33849 (11)	0.00276 (17)	0.70166 (8)	0.0292 (3)	
H10A	0.2699	0.0295	0.6894	0.044*	
H10B	0.3393	−0.0679	0.7445	0.044*	
H10C	0.3695	−0.0474	0.6585	0.044*	
H1	0.4346 (14)	0.975 (2)	0.4473 (9)	0.041 (5)*	

### Atomic displacement parameters (Å^2^)

**Table:**

	*U*^11^	*U*^22^	*U*^33^	*U*^12^	*U*^13^	*U*^23^
O1	0.0328 (6)	0.0288 (6)	0.0313 (5)	−0.0098 (4)	−0.0025 (4)	0.0006 (4)
O2	0.0286 (6)	0.0272 (5)	0.0317 (5)	−0.0030 (4)	−0.0064 (4)	0.0062 (4)
N1	0.0305 (7)	0.0222 (6)	0.0263 (6)	−0.0032 (5)	0.0027 (5)	0.0011 (5)
N2	0.0273 (7)	0.0236 (6)	0.0246 (6)	0.0019 (5)	−0.0012 (4)	−0.0018 (4)
N3	0.0231 (6)	0.0224 (6)	0.0233 (6)	−0.0019 (5)	−0.0001 (4)	−0.0014 (4)
C1	0.0267 (8)	0.0232 (7)	0.0248 (6)	−0.0018 (6)	0.0055 (5)	−0.0013 (5)
C2	0.0273 (8)	0.0226 (7)	0.0247 (6)	0.0026 (5)	0.0043 (5)	−0.0033 (5)
C3	0.0466 (10)	0.0244 (7)	0.0273 (7)	0.0038 (7)	−0.0018 (6)	−0.0007 (5)
C4	0.0192 (7)	0.0219 (7)	0.0247 (6)	0.0014 (5)	0.0039 (5)	0.0003 (5)
C5	0.0219 (7)	0.0243 (7)	0.0297 (7)	−0.0028 (5)	−0.0010 (5)	−0.0030 (5)
C6	0.0222 (7)	0.0310 (7)	0.0260 (7)	−0.0009 (6)	−0.0036 (5)	−0.0009 (6)
C7	0.0186 (7)	0.0243 (7)	0.0272 (7)	0.0025 (5)	0.0005 (5)	0.0019 (5)
C8	0.0241 (7)	0.0220 (7)	0.0283 (7)	−0.0004 (5)	−0.0018 (5)	−0.0022 (5)
C9	0.0235 (7)	0.0249 (7)	0.0242 (6)	0.0006 (5)	−0.0013 (5)	−0.0018 (5)
C10	0.0278 (8)	0.0254 (8)	0.0344 (7)	−0.0007 (6)	0.0007 (6)	0.0045 (6)

### Geometric parameters (Å, °)

**Table:**

O1—C1	1.2402 (17)	C4—C9	1.3833 (19)
O2—C7	1.3677 (16)	C4—C5	1.3999 (18)
O2—C10	1.4270 (17)	C5—C6	1.3845 (19)
N1—C2	1.3644 (18)	C5—H5	0.9300
N1—C1	1.3689 (18)	C6—C7	1.3946 (19)
N1—H1	0.938 (18)	C6—H6	0.9300
N2—C2	1.3014 (18)	C7—C8	1.3894 (18)
N2—N3	1.3997 (16)	C8—C9	1.3900 (19)
N3—C1	1.3689 (18)	C8—H8	0.9300
N3—C4	1.4226 (17)	C9—H9	0.9300
C2—C3	1.4816 (19)	C10—H10A	0.9600
C3—H3A	0.9600	C10—H10B	0.9600
C3—H3B	0.9600	C10—H10C	0.9600
C3—H3C	0.9600		
			
C7—O2—C10	117.08 (10)	C5—C4—N3	121.38 (12)
C2—N1—C1	108.50 (12)	C6—C5—C4	119.12 (12)
C2—N1—H1	126.5 (10)	C6—C5—H5	120.4
C1—N1—H1	125.0 (10)	C4—C5—H5	120.4
C2—N2—N3	104.21 (11)	C5—C6—C7	121.10 (12)
C1—N3—N2	111.38 (11)	C5—C6—H6	119.4
C1—N3—C4	129.40 (11)	C7—C6—H6	119.4
N2—N3—C4	119.21 (10)	O2—C7—C8	124.67 (12)
O1—C1—N3	128.40 (13)	O2—C7—C6	115.96 (11)
O1—C1—N1	127.64 (13)	C8—C7—C6	119.37 (12)
N3—C1—N1	103.96 (12)	C7—C8—C9	119.77 (12)
N2—C2—N1	111.95 (12)	C7—C8—H8	120.1
N2—C2—C3	125.14 (13)	C9—C8—H8	120.1
N1—C2—C3	122.88 (12)	C4—C9—C8	120.71 (12)
C2—C3—H3A	109.5	C4—C9—H9	119.6
C2—C3—H3B	109.5	C8—C9—H9	119.6
H3A—C3—H3B	109.5	O2—C10—H10A	109.5
C2—C3—H3C	109.5	O2—C10—H10B	109.5
H3A—C3—H3C	109.5	H10A—C10—H10B	109.5
H3B—C3—H3C	109.5	O2—C10—H10C	109.5
C9—C4—C5	119.91 (12)	H10A—C10—H10C	109.5
C9—C4—N3	118.71 (12)	H10B—C10—H10C	109.5
			
C2—N2—N3—C1	0.01 (14)	C1—N3—C4—C5	11.1 (2)
C2—N2—N3—C4	179.21 (11)	N2—N3—C4—C5	−167.89 (12)
N2—N3—C1—O1	179.91 (13)	C9—C4—C5—C6	−1.45 (19)
C4—N3—C1—O1	0.8 (2)	N3—C4—C5—C6	177.74 (12)
N2—N3—C1—N1	0.27 (14)	C4—C5—C6—C7	0.6 (2)
C4—N3—C1—N1	−178.83 (12)	C10—O2—C7—C8	−4.83 (19)
C2—N1—C1—O1	179.92 (14)	C10—O2—C7—C6	175.59 (12)
C2—N1—C1—N3	−0.44 (14)	C5—C6—C7—O2	−179.84 (12)
N3—N2—C2—N1	−0.31 (14)	C5—C6—C7—C8	0.6 (2)
N3—N2—C2—C3	−178.37 (13)	O2—C7—C8—C9	179.62 (12)
C1—N1—C2—N2	0.49 (16)	C6—C7—C8—C9	−0.8 (2)
C1—N1—C2—C3	178.61 (12)	C5—C4—C9—C8	1.2 (2)
C1—N3—C4—C9	−169.66 (13)	N3—C4—C9—C8	−178.00 (12)
N2—N3—C4—C9	11.31 (18)	C7—C8—C9—C4	−0.1 (2)

### Hydrogen-bond geometry (Å, °)

**Table:**

*D*—H···*A*	*D*—H	H···*A*	*D*···*A*	*D*—H···*A*
C10—H10C···O1^i^	0.96	2.57	3.4918 (18)	160
N1—H1···O1^ii^	0.938 (18)	1.825 (19)	2.7561 (16)	171.9 (16)

Symmetry codes: (i) *x*, *y*−1, *z*; (ii) −*x*+1, −*y*+2, −*z*+1.
